# Liver X receptor reduces proliferation of human oral cancer cells by promoting cholesterol efflux via up-regulation of ABCA1 expression

**DOI:** 10.18632/oncotarget.5428

**Published:** 2015-10-01

**Authors:** Tetsuharu Kaneko, Chihiro Kanno, Naoki Ichikawa-Tomikawa, Korehito Kashiwagi, Nanae Yaginuma, Chihiro Ohkoshi, Mizuko Tanaka, Takashi Sugino, Tetsuya Imura, Hiroshi Hasegawa, Hideki Chiba

**Affiliations:** ^1^ Department of Basic Pathology, Fukushima Medical University School of Medicine, Fukushima, Japan; ^2^ Division of Dentistry and Oral Surgery, Fukushima Medical University School of Medicine, Fukushima, Japan; ^3^ Department of Diagnostic Pathology, Shizuoka Cancer Center, Shizuoka, Japan

**Keywords:** LXR, nuclear receptor, squamous cell carcinoma, cell growth, metabolism

## Abstract

Liver X receptors (LXRs) contribute not only to maintain cholesterol homeostasis but also to control cell growth. However, the molecular mechanisms behind the LXR-mediated anti-proliferative effects are largely unknown. Here we show, by immunohistochemistry, that LXRα and LXRβ are differentially distributed in oral stratified squamous epithelia. By immunohistochemical and Western blot analyses, we also reveal that LXRα is abundantly expressed in human oral squamous cell carcinoma (HOSCC) tissues and cell lines. Cell counting, BrdU labeling and cell cycle assay indicated that LXR stimulation led to significant reduction of proliferation in HOSCC cells. Importantly, our study highlights, by using RNA interference, that the ATP-binding cassette transporter A1 (ABCA1)-accelerated cholesterol efflux is critical for the growth inhibitory action of LXRs in HOSCC cells. Moreover, we demonstrate that LXR activation reduces the growth of xenograft tumour of HOSCC cells in mice accompanied by the upregulation of ABCA1 expression and the decline of cholesterol levels in the tumour. These findings strongly suggested that targeting the LXR-regulated cholesterol transport, yielding in lowering intracellular cholesterol levels, could be a promising therapeutic option for certain types of cancers.

## INTRODUCTION

Cholesterol is indispensable not only for the biogenesis of cell membranes, bile acid or steroid hormones, but also for the control of cell proliferation [1]. Hypercholesterolemia promotes mammary tumour growth in mouse transgenic models [2], and epidemiologic studies show that a high-fat/high-cholesterol diet increases the risk of colorectal cancer [3, 4]. In addition, the levels of cholesterol in fibroblasts during S-phase of the cell cycle are known to be doubled compared with those during G1-phase [5]. Furthermore, cholesterol is generally accumulated in cancer tissues [6], and its depletion in prostate cancer leads to reduced proliferation and xenograft tumour growth [7]. Thus, limiting the circulating and intracellular cholesterol levels could be an encouraging therapeutic approach to treat cancer.

The liver X receptor α (LXRα; also known as NR1H3) and LXRβ (also known as NR1H2), members of the nuclear receptor superfamily, transcriptionally regulate the expression of a wide range of genes via forming heterodimers with the retinoid X receptor [8, 9]. Both LXRs are activated by cholesterol derivatives, including oxysterols and 24(*S*),25-epoxycholesterol, as well as synthetic agonists such as T0901317 and GW3965 [10–13]. LXRα is strongly expressed in metabolically active tissues such as the liver, intestine, kidney, skin, adrenal glands, adipose and macrophages, whereas LXRβ is ubiquitously distributed throughout the body [14].

LXRs play a key role in maintaining cholesterol homeostasis through control of the expression of various target genes involved in the uptake, storage, catabolism and transport of cholesterol [15–17]. In particular, they activate the expression of ATP-binding cassette transporter A1 (ABCA1), which accelerates cholesterol efflux resulting in the formation of HDL, and promote reverse cholesterol transport (RCT) from peripheral tissues to the liver [13, 18]. Mutations in the human *ABCA1* gene cause Tangier disease, in which patients exhibit little or no plasma HDL and prominent cholesterol deposition in peripheral tissues, indicating the functional relevance of ABCA1 in RCT [19–21]. Hence, the LXR-mediated RCT protects against cardiovascular diseases such as atherosclerosis.

In addition to cholesterol metabolism, LXRs participate in the regulation of cellular proliferation in many types of cells [22–24]. Their activation reduces proliferation of normal cells, including vascular smooth muscle cells, uterine endometrial cells, pancreatic β cells, hepatocytes, keratinocytes, and lymphocytes. Indeed, LXRα-null mice exhibit epithelial and stromal proliferation in ventral prostate [25], and LXRβ-deficient mice show marked splenomegaly due to lymphocyte expansion [26]. Moreover, LXR agonists decrease the proliferation of numerous tumour cells such as prostate, breast, ovarian, and colorectal cancer cells, as well as the growth of xenograft tumours in mice [23, 24]. However, the precise mechanism by which LXRs control cellular proliferation remains obscure.

We show in the present work that LXRα and LXRβ are distinctively expressed in both oral and skin epithelia along the base-to-surface axis. We also demonstrate that LXRα is greatly expressed in human oral squamous cell carcinoma (HOSCC) tissues and cell lines. Furthermore, we provide evidence showing that LXR activation diminishes the proliferation of HOSCC cells by enhancing cholesterol elimination through up-regulation of ABCA1 expression. In addition, we reveal that LXR stimulation slows down the growth of xenograft tumours of HOSCC cells in mice.

## RESULTS

### LXRα and LXRβ are differentially distributed in both oral and skin epithelia

**Figure 1:**
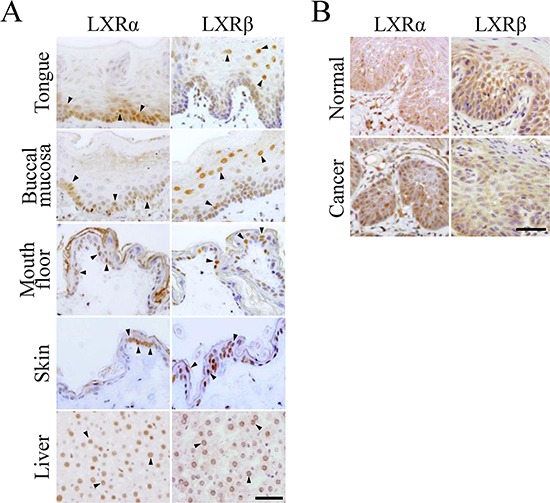
Expression of LXRα and LXRβ in normal epithelia and squamous cell carcinoma tissues of the oral cavity **A.** The indicated normal adult rat tissues were subjected to immunostaining with the corresponding antibodies. Arrowheads indicate LXRα- and LXRβ-positive signals in the nuclei. Scale bar, 100 μm. **B.** The human oral squamous cell carcinoma (HOSCC) and the surrounding normal tissues were immunostained with the corresponding antibodies. Scale bar, 100 μm.

Since the histological distribution of LXRα and LXRβ in oral and skin stratified squamous epithelia remains unclear, we first examined, by immunohistochemistry, their expression in normal rat tongue, buccal mucosa, mouth floor, and skin tissues (Figure 1A). LXRα was mainly observed in the nuclei of basal and parabasal cells in the rat oral epithelium, and the number of LXRα-positive cells was larger than that in the rat epidermis. On the other hand, LXRβ was strongly expressed in the nuclei of more differentiated prickle cells, and weakly or moderately detected in those of basal and parabasal cells. A similar expression pattern of LXRs was observed in human oral epithelium, although they were broadly distributed throughout the stratified layers compared with those in rats (Figure 1B). As expected, both LXRα and LXRβ were detected in the nuclei of rat hepatocytes as previously reported [8, 27]. Thus, LXRα and LXRβ levels were high in the proliferating cells and in more differentiated cells of the stratified squamous epithelia, respectively.

### LXRα is strongly expressed in HOSCC tissues and cell lines

We next evaluated, by immunohistochemistry, the expression of LXRα and LXRβ in HOSCC tissues resected from 12 patients (Figure 1B). The LXRα- and LXRβ-positive rates were significantly higher and lower than those in the surrounding normal oral tissues, respectively (Table 1). In addition, the percentage of cells expressing LXRα was markedly increased in 9 of 12 cases, and that of LXRβ was decreased in 11 of 12 cases.

**Table 1:** Positive expression of LXRα and LXRβ in HOSCC tissues

*Gender*	*Region*	*Positive expression* (*%*)			
		*LXRα*		*LXRβ*	
		*Normal*	*Cancer*	*Normal*	*Cancer*
F	tongue	53.5	53.5	36.9	19.3
F	tongue	49.2	52.7	28.6	14.7
M	tongue	35.5	33.1	40.0	29.9
M	tongue	12.4	48.5	22.0	15.5
M	tongue	15.6	25.0	60.3	45.1
M	tongue	47.0	61.0	67.6	65.6
M	gingiva	24.9	56.0	19.8	1.5
M	gingiva	27.6	44.8	26.2	8.6
F	gingiva	27.1	36.9	46.5	11.8
F	gingiva	30.2	45.2	33.5	11.2
M	oral floor	43.3	58.4	63.0	56.4
M	buccal mucosa	39.3	58.1	37.5	1.3
mean ± SD		33.8 ± 13.1	*47.8 ± 11.2	40.2 ± 16.1	*23.4 ± 21.3

The HOSCC and the surrounding normal tissues were obtained from 12 patients, and the percentage of their positive staining for LXRs was determined. The values represent the mean ± SD.

* *P* < 0.05.

**Figure 2:**
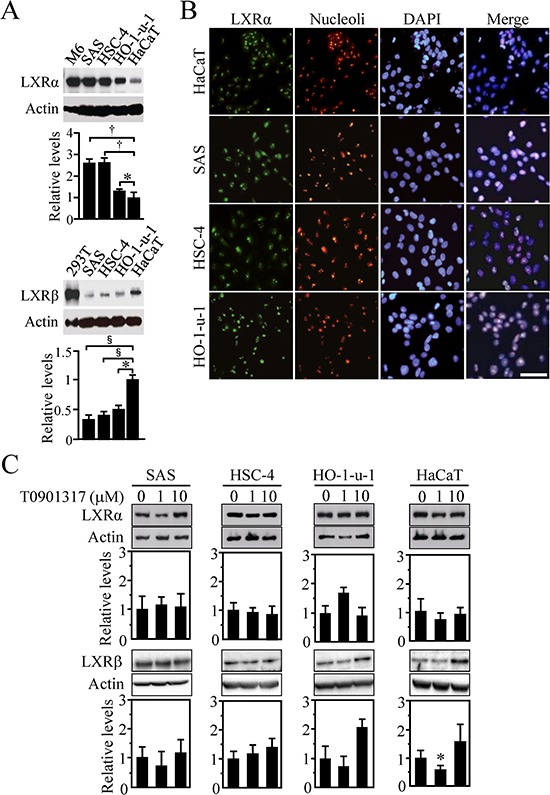
Expression of LXRα and LXRβ in human oral squamous cell carcinoma (HOSCC) cells **A.** Western blot analysis for LXRs was performed in the indicated cell lines. Rat liver cells (M6), LXRβ-overexpressed 293T cells and the human skin-derived cell line HaCaT were used as controls. Quantification of the protein levels is shown in the histograms. The values represent the mean ± SD (*n* = 4). **B.** Cells were stained for LXRα together with the nucleoli marker and the nuclear marker DAPI. Scale bar, 50 μm. **C.** Effect of T0901317 on LXRα and LXRβ expression in the indicated cell lines. Cells treated for 72 h with the vehicle or T0901317 (1 and 10 μM) were subjected to Western blot for the indicated molecules. Quantification of the protein levels is shown in the histograms. The values represent the mean ± SD (*n* = 5). All statistic values, *P < 0.05, ^†^*P* < 0.01, ^§^*P* < 0.001.

We also investigated, by Western blot analysis, the expression levels of LXRs in HOSCC cell lines (SAS, HSC-4, and HO-1-u-1) using rat liver cells (M6), LXRβ-overexpressed 293T cells and a human skin-derived cell line (HaCaT) as controls (Figure 2A). As expected, the amount of LXRα and LXRβ protein in the HOSCC cell lines was significantly greater and smaller than that in the HaCaT cells, respectively. Moreover, LXRα was often observed in nucleoli of both HOSCC cells (Figure 2B) and normal oral tissues (Figures 1A and 1B) as previously reported [28].

Since the expression of LXRα in human skin fibroblasts and macrophages is autoregulated by LXR agonists [29–31], we subsequently examined whether this was the case for the HOSCC and HaCaT cells. However, expression levels of LXRα protein were not largely altered in these cells treated for 72 h with 1 or 10 μM T0901317 (Figure 2C) as reported in primary human adipocytes, hepatocytes or a human intestinal cell line [30]. In addition, no induction of LXRβ expression was observed in the cells after T0901317 treatment except the 1 μM T0901317-exposed HaCaT cells, in which the LXRβ amount was rather decreased.

### LXR activation reduces cellular growth in HOSCC cells

**Figure 3:**
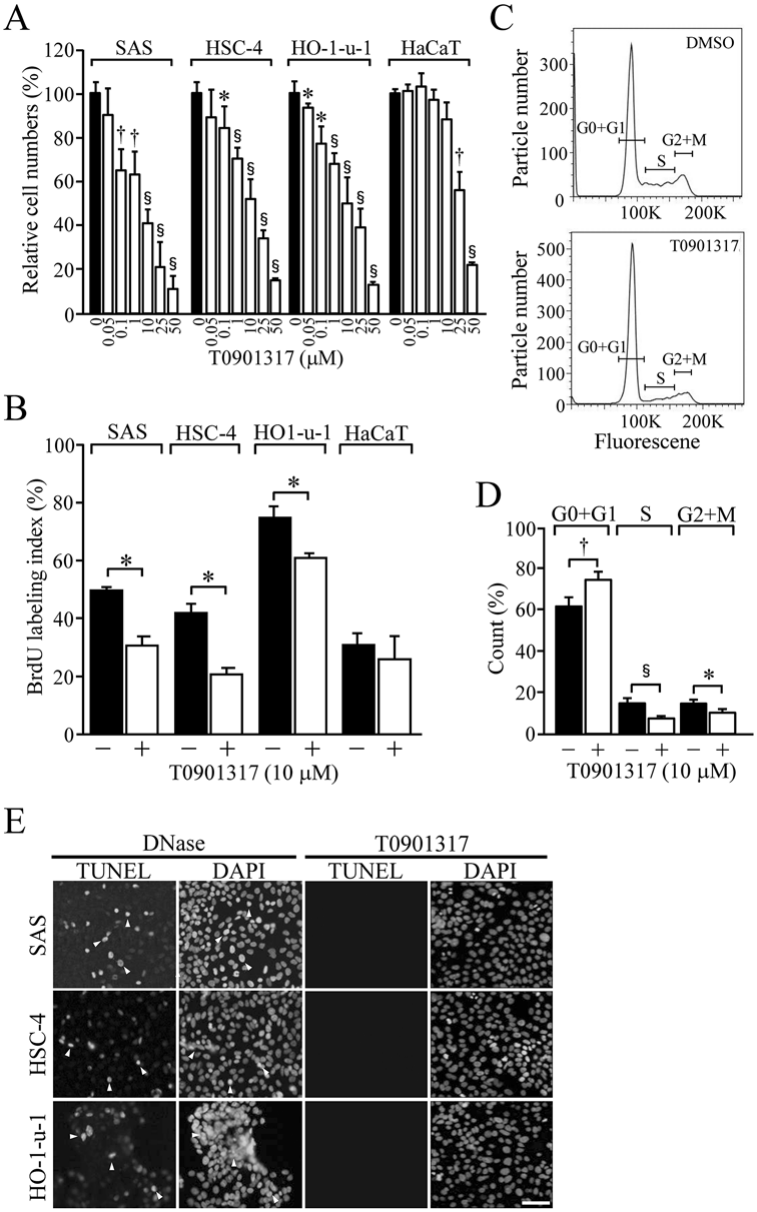
The effect of T0901317 on cellular proliferation and apoptosis in HOSCC cell lines **A.** The viable cell number was counted after treating the indicated cells for 48 h with the vehicle or T0901317 (0.05, 0.1, 1, 10, 25, and 50 μM). Quantification of the relative cell number is shown in the histograms. The values represent the mean ± SD (*n* = 3). **B.** The indicated cells were grown for 72 h in the presence or absence of 10 μM T0901317, and the BrdU-labeling index was determined. The values represent the mean ± SD (*n* = 4). **C.** The cell cycle profile in SAS cells grown as in B. **D.** The proportion of SAS cells in cell cycle phases. The values represent the mean ± SD (*n* = 5). **E.** Cells were exposed for 72 h with 10 μM T0901317, and subjected to TUNEL assay together with DAPI staining. As a positive control, cells were treated with DNase. Arrowheads indicate the positive signals in the nuclei of apoptotic cells. Scale bar, 50 μm. All statistic values, *P < 0.05, ^†^*P* < 0.01, ^§^*P* < 0.001.

Because LXRα was abundantly expressed in HOSCC cells and tissues, we next investigated whether the synthetic LXR agonist T0901317 treatment affected cell viability and proliferation of HOSCC cell lines. The viable cell numbers after 48-h T0901317 treatment were significantly decreased in HO-1-u-1 cells at 0.05, 0.1, 1, 10, 25 and 50 μM, as well as in HSC-4 and SAS cells at 0.1 μM and higher concentrations (Figure 3A). In marked contrast, the numbers of HaCaT cells were not affected by exposure to 0.05–10 μM T0901317 but to 25 and 50 μM. The relative cell numbers of SAS, HSC-4, HO-1-u-1 and HaCaT cells treated for 48 h with 10 μM T0901317 were 41.6 ± 7.4%, 52.3 ± 8.7%, 49.9 ± 12.4%, and 87.7 ± 8.9%, respectively. In addition, trypan blue-stained cells were scarcely observed at this concentration (data not shown). Furthermore, another synthetic LXR ligand GW3965 reduced the numbers of SAS cells in a dose-dependent manner, whereas it marginally influenced those of HaCaT cells (Supplementary Figure S1).

Since T0901317 strikingly reduced the cell numbers of the HOSCC cell lines, we next determined whether cell proliferation and/or death contributed to these effects. On BrdU assay, cellular proliferation was significantly decreased in SAS, HSC-4 and HO-1-u-1 cells exposed for 72 h to 10 μM T0901317, while it was barely affected in HaCaT cells (Figure 3B). We also analysed the cell cycle profile in SAS cells treated for 72 h with either the vehicle or 10 μM T0901317 (Figure 3C). The percentage of cells in G0 and G1 phases was significantly increased in T0901317-exposed cells compared with that in vehicle-treated cells (Figure 3D).

Because LXR stimulation is known to induce apoptosis in ovarian and prostate cancer cells [32, 33], we subsequently evaluated, by the TUNEL assay, the effects of T0901317 on apoptosis in HOSCC cell lines. As shown in Figure 3E, apoptotic cells were hardly detected in these HOSCC cell lines treated for 72 h with 10 μM T0901317. Taken collectively, these results indicated that T0901317 reduced the viable cell numbers in HOSCC cells through inhibition of cellular proliferation.

### LXR activation decreases proliferation of HOSCC cells by promoting cholesterol efflux via induction of ABCA1 expression

**Figure 4:**
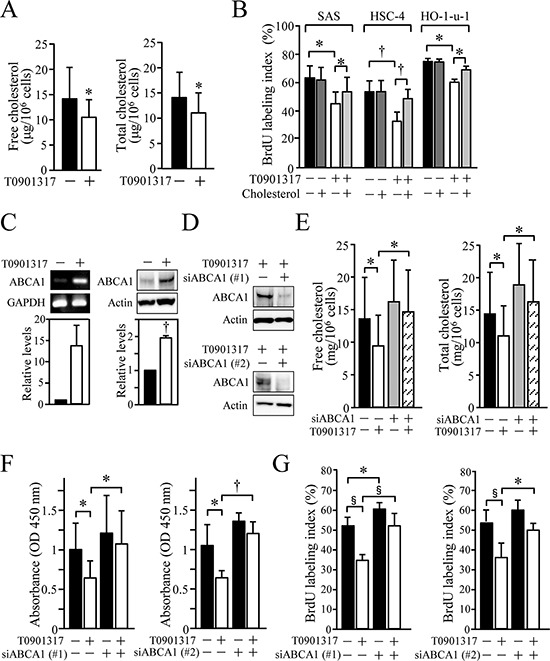
The significance of ABCA1-mediated cholesterol efflux for the anti-proliferative effect of T0901317 in HOSCC cell lines **A.** SAS cells were grown for 72 h in the presence or absence of 10 μM T0901317, and their free and total cholesterol levels were measured. The values represent the mean ± SD (*n* = 8). **B.** SAS, HSC-4 and HO-1-u-1 cells were treated for 72 h with 10 μM T0901317 and/or 2 mg/ml cholesterol, and their BrdU-labeling index (%) was calculated. The values represent the mean ± SD (*n* = 3). **C.** SAS cells were cultured as in A, and the ABCA1 expression was determined by RT-PCR and Western blot analyses. The mRNA and protein levels were normalised to the corresponding GAPDH and actin levels, respectively, and expressed relative to the amount present in cells grown in the absence of T0901317, which was taken as 1. The values for ABCA1 mRNA represent the mean ± SD (*n* = 3). **D.** SAS cells were transfected with negative control siRNA or siRNA against ABCA1, and exposed for 36 h to 10 μM T0901317. Western blot analysis was performed, and the protein levels were expressed as in C. **E.** SAS cells were transfected as in D and treated for 72 h with 10 μM T0901317. The cholesterol levels were measured and expressed as the mean ± SD (*n* = 6). (F, G) SAS cells were transfected with negative control siRNA or two distinct siRNAs against ABCA1, and cultured as in E. XTT **F.** and BrdU **G.** assays were performed. Quantification of the relative cell number is shown in the histograms, and the values represent the mean ± SD (F, *n* = 9; G, *n* = 6). All statistic values, *P < 0.05, ^†^*P* < 0.01, ^§^*P* < 0.001.

To verify whether cholesterol metabolism participates in the anti-proliferative activity of the LXR agonist, we next determined the amount of cholesterol in SAS cells grown in the presence or absence of T0901317. As shown in Figure 4A, the levels of free and total cholesterol were significantly reduced in SAS cells treated for 72 h with 10 μM T0901317 (Figure 4A). It is noteworthy that, upon cholesterol addition, anti-proliferative effects of T0901317 on the three kinds of HOSCC cell lines were significantly hindered (Figure 4B), strongly suggesting that the LXR ligand reduced their cellular growth by decreasing the intracellular cholesterol levels.

Among LXR target genes involved in cholesterol homeostasis, we next focused on ABCA1 and determined whether it was responsible for the anti-proliferative effect of T0901317 in HOSCC cells. As expected, the expression of ABCA1 mRNA and protein was induced in SAS cells exposed for 72 h to 10 μM T0901317 (Figure 4C). When the expression of ABCA1 was suppressed in the cells by the siRNA, reduction of the intracellular cholesterol amounts after T0901317 treatment was significantly prevented (Figures 4D and 4E). More importantly, the knockdown of ABCA1 expression using two distinct siRNAs significantly reversed the anti-proliferative effects of T0901317 in SAS cells (Figures 4F and 4G). Hence, ABCA1 primarily contributed to the anti-proliferative effects of the synthetic LXR ligand, though other LXR target genes such as ABCG1 may also be involved in the effects.

### LXR activation reduces the growth of xenograft tumour of HOSCC cells in mice

**Figure 5:**
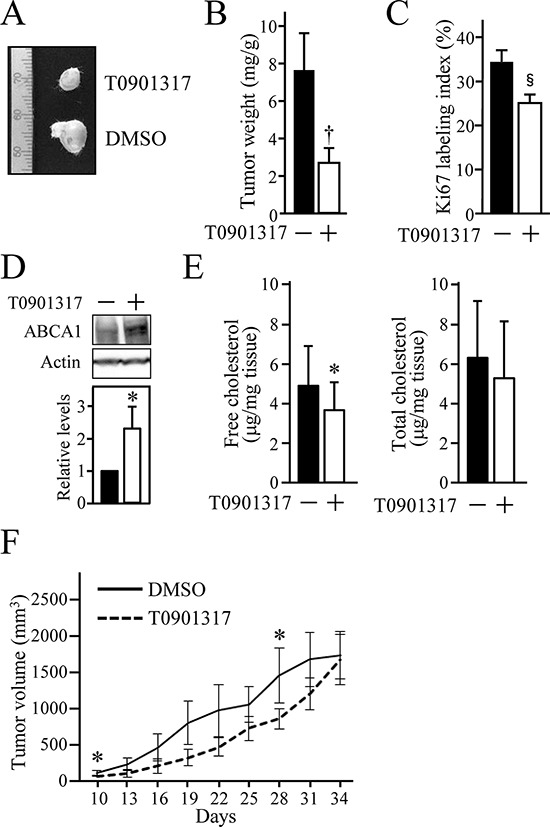
The anti-tumour effect of T0901317 on xenografts of HOSCC cells in mice SCID mice were subcutaneously inoculated on their backs or chests with SAS cells, and intraperitoneally treated with the vehicle (DMSO) or T0901317 (10 μg/g mice) every three days. At 15 days after the first treatment, the tumour was resected and subjected to the further analyses **A–E.** (A) The representative appearance of xenografted tumours. (B) The tumour weight was measured and expressed as mg/1g of mouse body weight. The values represent the mean ± SD (*n* = 8). (C) Cell proliferation of SAS xenografts was evaluated by the Ki67 index. The values represent the mean ± SD (*n* = 8). (D) The expression of ABCA1 protein in xenografts was determined by Western blot analysis. The protein levels were normalised to the corresponding actin levels, and expressed relative to the amount present in tumours treated with the vehicle, which was taken as 1. (E) The cholesterol levels in xenograft tumour were measured and expressed as μg/1 mg of tumour tissue weight. The values represent the mean ± SD (*n* = 8). **F.** The tumour size was measured every three days as indicated. The values represent the mean ± SD (*n* = 5). All statistic values, *P < 0.05, ^†^*P* < 0.01.

To validate whether LXR stimulation also leads to growth inhibition of HOSCC cells *in vivo* with a similar mechanism to that observed *in vitro*, we subsequently clarified the effect of T0901317 on the growth of xenograft tumour of HOSCC cells. The tumour weight in SCID mice inoculated with SAS cells was significantly decreased in mice treated for 15 days with T0901317 (10 μg/g mice) (Figures 5A and 5B). The Ki67-labeling index of tumour cells was also significantly reduced in mice exposed for 15 days to T0901317 (Figure 5C). In addition, treatment with T0901317 resulted in the upregulation of ABCA1 expression and the decline of cholesterol levels in the xenograft tumour (Figures 5D and 5E). Furthermore, the tumour volume of the T0901317-exposed mice was decreased to about half at days 10, 13, 16, 19 and 22, and to approximately 2/3 to 3/4 at days 25, 28 and 31 compared with that of the DMSO-treated mice, although no statistical significance was detected except for days 10 and 28 (Figure 5F). However, there was no difference in the tumour volume at day 34 between the two groups, probably due to the action mechanism of T090137 that does not inhibit but slow down cell proliferation.

Mice that received T0901317 (10 μg/g mice) treatment every three days for 15 days showed no serious adverse events, and their body weight was similar to that in the control group (Supplementary Figure S2A). There were also no significant differences in serum free, LDL and HDL cholesterol levels between the two groups (Supplementary Figure S2B–S2E). By contrast, serum triglyceride and free cholesterol amount in the T0901317-treated mice was significantly increased compared with that in the control group (Supplementary Figure S2F), although fatty liver was not observed in the mice (data not shown).

## DISCUSSION

In the present study, we demonstrated that the distribution pattern of LXRα was different from that of LXRβ in both oral and skin stratified squamous epithelia. LXRα was strongly expressed in the proliferating basal and parabasal cells, whereas LXRβ expression predominated in more differentiated prickle cells. Although LXRα was reported to be expressed throughout the layers of human epidermis [34], its expression seems to be rather obvious in the basal layer from what we observed in their presented data. The distinct localisation of LXRα and LXRβ is also evident in the intestine and brain [35–38]. For instance, LXRα mRNA is expressed in the villus epithelium of the ileum and the surface epithelium of the colon, while LXRβ is ubiquitously localised along the crypt/villus (surface mucosa) axis [36]. Taken collectively, our results reinforce the notion that each LXR subtype possesses specific physiological functions.

The second conclusion of our study is that LXRα protein is abundantly expressed in HOSCC tissues. The aberrant expression of LXRα was confirmed in three kinds of HOSCC cell lines. Taken together with results showing that LXRβ expression was strikingly diminished in HOSCC tissues and cell lines, it is likely that the anti-proliferative effects of T0901317 in the cells are mediated by the LXRα subtype. The significant upregulation of LXRα mRNA is also observed in freshly isolated B cells from 10 patients with chronic lymphoblastic leukemia compared with those from healthy donors [39]. Similarly, LXRα is the major subtype in three of four human prostate cancer cell lines and in two of two human colon adenocarcinoma cell lines [40, 41]. In contrast, LXRβ is the major subtype expressed in melanoma and pancreatic cancers [42, 43]. In either case, it should be noted that LXRα and/or LXRβ are overexpressed in various types of cancers compared with the corresponding normal tissues and cells.

The most important conclusion of the present work is that LXR activation reduces proliferation of HOSCC cells by enhancing cholesterol efflux via activation of ABCA1 expression. This conclusion was drawn from the following results; 1) Cholesterol addition reversed the anti-proliferative effects of T0901317 on three types of HOSCC cell lines, 2) The knockdown of T0901317-induced ABCA1 expression prevented the influence of T0901317 that diminished the intracellular cholesterol levels and cellular growth in HOSCC cells. In this respect, Yvan-Charvet et al. reported that the proliferation of hematopoietic stem and progenitor cells in the bone marrow is regulated by a similar mechanism [44]. They demonstrated that exposure of bone marrow myeloid cells to T0901317 led to increased cholesterol efflux and decreased growth factor-stimulated proliferation in wild-type cells but not in *Abca1/Abcg1*-deficient cells. In addition, LXRβ restricts premature expansion of T cells by restricting cellular cholesterol levels via activation of ABCA1 expression [45]. Concerning malignant cells rather than normal cells, the significance of ABCA1 in the control of cellular proliferation is revealed by using a human prostate cancer cell line LNCaP [40]. On the other hand, LXRβ stimulation suppresses the growth, invasion and metastasis of various melanoma cell lines by activating apolipoprotein-E (ApoE) expression but affects neither cell proliferation nor viability rates [43]. It should also be mentioned that T0901317 or GW3965 treatment significantly decreased the cellular proliferation of the HOSCC cell lines at concentrations that hardly affected the human skin-derived keratinocyte cell line HaCaT. Hence, at the least, certain types of cancers could be treated by targeting the LXR-regulated cholesterol transport.

Furthermore, we showed that LXR stimulation decreased the growth of xenograft tumour of HOSCC cells in mice. In addition, LXR activation resulted in the upregulation of ABCA1 expression and the reduction of cholesterol levels in the xenograft tumour, suggesting that LXR exhibits anti-proliferative effects *in vivo* with the machinery similar to that observed *in vitro*. Another issue that should be discussed is undesirable side effects of the synthetic LXR agonists in the whole body. Mice that were treated with T0901317 for 15 days displayed elevated plasma triglyceride and free cholesterol levels, which is in good agreement with previous results [11, 46]. The alternative pan-LXR agonist GW3965 also raises plasma triglyceride amount [47]. Therefore, the development of novel LXR agonists is required for treating cancer with minimised side effects. It should also be verified as to whether they are effective in preventing tumour growth even in long-term administration, because the anti-tumour effects of T0901317 were not detected in HOSCC xenografts at day 34.

In summary, we have provided evidence showing that LXR acts as a growth-inhibitor to activate ABCA1 expression and stimulate cholesterol efflux in HOSCC cells. To the best of our knowledge, this study is the first report presenting that the ABCA1-facilitated cholesterol elimination is responsible for the anti-proliferative function of LXR in cancer cells. The possible involvement of cholesterol efflux via the LXR/ABCA1 pathway in growth inhibition and the potential therapeutic efficacy of novel LXR agonists should be examined in a variety of cancers.

## MATERIALS AND METHODS

### Animal and human studies

Liver, skin and oral cavity tissues were obtained from 24-week-old male Wister rats (Japan SLC, Shizuoka, Japan). Tissues were fixed with 4% paraformaldehyde and embedded in paraffin. HOSCC tissues were obtained from 12 adult patients whose primary tumour was resected by surgical operation at Fukushima Medical University. The primary regions were tongue (6 cases), gingiva (4 cases), buccal mucosa (1 case) and oral floor (1 case), and informed consent was obtained from all patients. Human tissues were fixed with 10% neutral formalin and paraffin-embedded.

For xenograft experiments, SAS cells (1 × 10^6^ cells) were subcutaneously inoculated into the backs or chests of 8- to 10-week-old SCID mice (CLEA, Tokyo, Japan). From one day after injection, the mice were intraperitoneally treated with a vehicle (DMSO) or T0901317 (10 μg/g mice) every three days. The tumour volume (mm^3^) was calculated by the ellipse formula (*V* = 4/3πabc).

All these experiments were approved by the Experimental Animal Center and the Ethical Committee of Fukushima Medical University and carried out in accordance with regulations on animal and human experiments.

### Cell culture

Three HOSCC cell lines (SAS, HSC-4 and HO-1-u-1) were supplied by Riken BioResource Center Cell Bank (Ibaraki, Japan). The rat liver cell line M6 (Fukushima Medical University, Department of Basic Pathology) and the human skin-derived cell line HaCaT (supplied by Juntendo University) were used as controls. Cells were cultured at 37°C under 5% CO_2_ in RPMI 1640 (WAKO, Tokyo, Japan) except for HaCaT cells, which were grown in Dulbecco's modified Eagle's medium high glucose (Sigma Aldrich, Saint Louis, USA). Medium was supplemented with 10% fetal bovine serum, 100 U/ml penicillin, 100 μg/ml streptomycin and 0.25 μg/ml amphotericin B (Life Technologies, Carlsbad, USA).

### Reagents and antibodies

T0901317 (Alexis Biochemicals, Lausen, Switzerland) and GW3956 (Sigma Aldrich), synthetic agonists for both LXRα and LXRβ, were diluted with DMSO (Sigma Aldrich,). The cell lysates of LXRβ-overexpressed 293T cells were obtained from Santa Cruz Biotechnology (Dallas, USA). Water-soluble cholesterol was obtained from Sigma Aldrich. Mouse monoclonal antibodies (mAbs) against LXRα, Ki67 and β-actin were purchased from Perseus Proteomics (Tokyo, Japan), NeoMarkers (Fremont, USA) and Sigma Aldrich, respectively. For a nucleoli marker, a mouse mAb generated by using human Raji cell nuclei as an antigen, was obtained from Millipore (Massachusetts, USA). A goat polyclonal antibody (pAb) against LXRβ and a rabbit pAb against ABCA1 were purchased from Santa Cruz Biotechnology and Abcam (Tokyo, Japan), respectively. A rat mAb against BrdU was purchased from Serotec (Oxford, UK).

### Immunohistochemistry

For immunostaining of LXRα, LXRβ and Ki67, paraffin-embedded tissue sections were deparaffinised in xylene and rehydrated through graded ethanol. Antigen retrieval was subsequently performed by autoclaving for 15 min at 121°C in sodium citrate buffer (pH 6.0). After blocking with 5% skimmed milk (Morinaga Milk Industry, Tokyo, Japan), the sections were incubated with primary antibodies overnight at 4°C. After washing, immunostaining was performed by using Histofine Simple Stain MAX-PO (MULTI) Kit (Nichirei, Tokyo, Japan) and DAB as a chromogen according to the manufacturer's instructions. At least 1000 cells per section were examined in three randomly selected high-power fields, and the percentage of positive nuclear staining was calculated.

For immunofluorescence staining, cells grown on 4-well chamber slides (BD Biosciences, San Jose, USA) were fixed in 4% paraformaldehyde and permeabilised with 0.1% Triton X-100 for 10 min. For HO-1-u-1 cells, the slides were coated with 100 μg/ml of calf skin collagen (Elastin Product Company, Owensville, USA). After blocking with 5% skimmed milk in PBS for 20 min, they were incubated for 1 h at room temperature with mouse anti-LXRα mAb, and rinsed again with PBS, followed by reaction for 45 min at room temperature with FITC-labeled anti-mouse IgG (Amersham Biosciences, Bucks, UK). After washing three times with PBS, the cells were fixed with 2% paraformaldehyde again. The slides were then incubated with mouse anti-nucleoli mAb for 1 h, and reacted for 45 min at room temperature with rhodamine-labeled anti-mouse IgG (GE Healthcare Bio-Sciences, Pittsburgh, USA). They were then mounted using a PloLong Gold antifade reagent with DAPI (Invitrogen, New York, USA).

### Western blot analysis

Cells and tissues were washed twice with PBS, and homogenised in CelLytic MT (Sigma Aldrich) containing a protease inhibitor (Complete mini EDTA-free; Roche Diagnostics, Mannheim, Germany) using a 27-gauge needle and syringe. After being centrifuged for 30 min at 4°C at 15,000 × *g*, cell lysates were resolved by SDS-PAGE and electrophoreticallly transferred onto a polyvinylidene difluoride membrane (Immobilon; Millipore). The membrane was saturated with tris-buffered saline containing 10% skimmed milk and 0.05% Tween 20, and incubated overnight at 4°C with primary antibodies against LXRα, LXRβ or ABCA1. After washing, they were incubated for 1 h at room temperature with horseradish peroxidase-labeled anti-mouse (GE Healthcare Bio-Sciences), anti-rabbit (GE Healthcare Bio-Sciences) or anti-goat IgG (Dako, Glostrup, Denmark). The blots were stripped with Restore Western Blot Stripping Buffer (Pierce, Rockford, USA) according to the manufacturer's instructions, and immunoprobed with an anti-actin Ab. Signals in immunoblots were quantified by using Image-Pro Plus software (Nippon Roper, Tokyo, Japan).

### Cell counting and XTT assay

Cells were plated on a 6-well culture plate at 5 × 10^5^ cells per well, grown for 24 h, and treated for 48 h with the vehicle (DMSO) or T0901317 (0.05, 0.1, 1, 10, 25 and 50 μM). They were stained with 0.3% trypan blue, and the number of viable cells was counted by using a Bürker-Türk hemocytometer.

XTT assay was performed using a Cell Proliferation Kit II (XTT; Roche Diagnostics) according to the manufacturer's protocol.

### Cell proliferation assay

For analysis of DNA synthesis, cells grown on 4-well chamber slides were exposed for 72 h to 10 μM T0901317 and/or 1 mg/ml cholesterol. They were incubated for 60 min with 10 μM BrdU (Sigma Aldrich), fixed in 4% paraformaldehyde, and permeabilised with 0.1% Triton X-100. They were subsequently incubated with 2 N HCl for 30 min at 37°C and with 0.1 M borate buffer (pH 8.5) for 10 min at room temperature. After blocking with 2% BSA, they were incubated with an anti-BrdU antibody overnight at 4°C, followed by reaction with FITC-labeled anti-rat IgG. At least 300 cells per slide were examined in three randomly selected high-power fields, and the percentage of positive staining was calculated.

The cell cycle profile was determined by flow cytometry based on cellular DNA content by using a Cell Cycle Phase Determination Kit II (Cayman Chemicals, Ann Arbor, USA). Subconfluent cultures of the vehicle- or T0901317-treated cells were grown for 24 h in serum-free medium, and 1 × 10^6^ cells were incubated with 0.1% propidium iodide (Sigma Aldrich) for 30 min at room temperature. They were analysed by using a flow cytometer (CantoII; BD Biosciences).

### Apoptosis assay

For the TUNEL assay, cells cultured on 4-well chamber slides were treated for 72 h with 10 μM T0901317, and apoptotic cells were detected by using an In Situ Cell Death Detection Kit (Roche Diagnostics) according to the manufacturer's instruction. As a positive control, cells were treated with 3 U/ml DNase (TURBO DNA-free; Ambion, Austin, USA). Cell nuclei were counterstained with DAPI.

### Microscopy

All samples were examined by using flexible upright fluorescence microscope (BX61; OLYMPUS, Tokyo, Japan), and photomicrographs were recorded by using DP controller software (OLYMPUS).

### Small interfering RNA and transfection

siRNA oligonucleotides against human ABCA1 (AF275948.1) were obtained from Life Technologies, and negative control siRNAs (Mission_Negative control SIC-001) were purchased from Sigma-Aldrich. The target sequences were as follows: siABCA1 #1, sense (5′-GGUCAAACUUGAAGCUUCAAGAUU U-3′) and antisense (5′-AAAUCUUGAAGCUUCAAGUUUGAG C-3′); siABCA1 #2, sense (5′-CAGUACACAUUUGUCAG CAAUGAU G-3′) and antisense (5′-CAUCAUUGCU GACAAAUGUGUACU G-3′). SAS cells (1 × 10^6^ cells) were transfected with 500 pmol siRNAs by using a Gene Pulser II (Bio-Rad, Hercules, USA), resuspended in complete medium, and seeded in 10-cm culture dish. Twelve h after transfection, they were followed by culture in the presence or absence of T0901317.

### RT-PCR

Total RNA was isolated using TRIzol reagent (Invitrogen). Reverse transcription and subsequent PCR were performed by using a SuperScript Ш First-Standard Synthesis System (Invitrogen) and an Advantage cDNA PCR Kit (Clonetech, Mountain View, USA) according to the manufacturer's instructions. The PCR primers for human ABCA1 were 5′-AACAGTTTGTGGCCCTTTTG-3′ and 5′-AGTTCCAGGCTGGGGTACTT-3′. PCR was performed at 30 cycles, and the expression of GAPDH mRNA was checked as the internal control. Aliquots of PCR products were loaded onto 1% agarose gel, and analysed by ImageQuant LAS 4000 (GE Healthcare Bio-Sciences) after staining with ethidium bromide.

### Plasma lipid content

Serum cholesterol and triglycerides were measured by enzyme-linked immunosorbent assay.

### Statistical analyses

Statistical significance of differences was evaluated by the Student's *t*-test and the Wilcoxon signed-rank test, and analysed by a StatMate software (ATM, Tokyo, Japan) and SSPS software (IBM. USA).

## SUPPLEMENTARY MATERIAL FIGURES



## References

[1] Simons K IE (2000). How cells handle cholesterol. Science.

[2] Silvente-Poirot S, Poirot M (2014). Cancer. Cholesterol and cancer, in the balance. Science.

[3] Giovannucci E, Michaud D (2007). The role of obesity and related metabolic disturbances in cancers of the colon, prostate, and pancreas. Gastroenterology.

[4] Yasuda T, Grillot D, Billheimer JT, Briand F, Delerive P, Huet S, Rader DJ (2010). Tissue-specific liver X receptor activation promotes macrophage reverse cholesterol transport *in vivo*. Arteriosclerosis, thrombosis, and vascular biology.

[5] Singh P, Saxena R, Srinivas G, Pande G, Chattopadhyay A (2013). Cholesterol biosynthesis and homeostasis in regulation of the cell cycle. PloS one.

[6] Murai T (2015). Cholesterol lowering: role in cancer prevention and treatment. Biological chemistry.

[7] Yue S, Li J, Lee SY, Lee HJ, Shao T, Song B, Cheng L, Masterson TA, Liu X, Ratliff TL, Cheng JX (2014). Cholesteryl ester accumulation induced by PTEN loss and PI3K/AKT activation underlies human prostate cancer aggressiveness. Cell metabolism.

[8] Apfel R, Benbrook D, Lernhardt E, Ortiz MA, Salbert G, Pfahl M (1994). A novel orphan receptor specific for a subset of thyroid hormone-responsive elements and its interaction with the retinoid/thyroid hormone receptor subfamily. Molecular and cellular biology.

[9] Willy PJ, Umesono K, Ong ES, Evans RM, Heyman RA, Mangelsdorf DJ (1995). LXR, a nuclear receptor that defines a distinct retinoid response pathway. Genes & development.

[10] Janowski BA, Willy PJ, Devi TR, Falck JR, Mangelsdorf DJ (1996). An oxysterol signalling pathway mediated by the nuclear receptor LXR alpha. Nature.

[11] Schultz JR, Tu H, Luk A, Repa JJ, Medina JC, Li L, Schwendner S, Wang S, Thoolen M, Mangelsdorf DJ, Lustig KD, Shan B (2000). Role of LXRs in control of lipogenesis. Genes & development.

[12] Collins JL, Fivush AM, Watson MA, Galardi CM, Lewis MC, Moore LB, Parks DJ, Wilson JG, Tippin TK, Binz JG, Plunket KD, Morgan DG, Beaudet EJ, Whitney KD, Kliewer SA, Willson TM (2002). Identification of a nonsteroidal liver X receptor agonist through parallel array synthesis of tertiary amines. Journal of medicinal chemistry.

[13] Calkin AC, Tontonoz P (2012). Transcriptional integration of metabolism by the nuclear sterol-activated receptors LXR and FXR. Nature reviews Molecular cell biology.

[14] Lu TT, Repa JJ, Mangelsdorf DJ (2001). Orphan nuclear receptors as eLiXiRs and FiXeRs of sterol metabolism. The Journal of biological chemistry.

[15] Repa JJ, Turley SD, Lobaccaro JA, Medina J, Li L, Lustig K, Shan B, Heyman RA, Dietschy JM, Mangelsdorf DJ (2000). Regulation of absorption and ABC1-mediated efflux of cholesterol by RXR heterodimers. Science.

[16] Zelcer N, Hong C, Boyadjian R, Tontonoz P (2009). LXR regulates cholesterol uptake through Idol-dependent ubiquitination of the LDL receptor. Science.

[17] Zelcer N, Tontonoz P (2006). Liver X receptors as integrators of metabolic and inflammatory signaling. The Journal of clinical investigation.

[18] Rader DJ, Tall AR (2012). The not-so-simple HDL story: Is it time to revise the HDL cholesterol hypothesis?. Nature medicine.

[19] Bodzioch M, Orso E, Klucken J, Langmann T, Bottcher A, Diederich W, Drobnik W, Barlage S, Buchler C, Porsch-Ozcurumez M, Kaminski WE, Hahmann HW, Oette K, Rothe G, Aslanidis C, Lackner KJ (1999). The gene encoding ATP-binding cassette transporter 1 is mutated in Tangier disease. Nature genetics.

[20] Brooks-Wilson A, Marcil M, Clee SM, Zhang LH, Roomp K (1999). Mutation in ABC1 in Tangier disease and familial high-density lipoprotein deficiency. Nature genetics.

[21] Rust S, Rosier M, Funke H, Real J, Amoura Z, Piette JC, Deleuze JF, Brewer HB, Duverger N, Denefle P, Assmann G (1999). Tangier disease is caused by mutations in the gene encoding ATP-binding cassette transporter 1. Nature genetics.

[22] Jamroz-Wisniewska A, Wojcicka G, Horoszewicz K, Beltowski J (2007). Liver X receptors (LXRs). Part II: non-lipid effects, role in pathology, and therapeutic implications. Postepy higieny i medycyny doswiadczalnej.

[23] Bovenga F, Sabba C, Moschetta A (2015). Uncoupling Nuclear Receptor LXR and Cholesterol Metabolism in Cancer. Cell metabolism.

[24] Lin CY, Gustafsson JA (2015). Targeting liver X receptors in cancer therapeutics. Nature reviews Cancer.

[25] Kim HJ, Andersson LC, Bouton D, Warner M, Gustafsson JA (2009). Stromal growth and epithelial cell proliferation in ventral prostates of liver X receptor knockout mice. Proceedings of the National Academy of Sciences of the United States of America.

[26] Gabbi C, Warner M, Gustafsson JA (2009). Minireview: liver X receptor beta: emerging roles in physiology and diseases. Molecular endocrinology.

[27] Teboul M, Enmark E, Li Q, Wikstrom AC, Pelto-Huikko M, Gustafsson JA (1995). OR-1, a member of the nuclear receptor superfamily that interacts with the 9-cis-retinoic acid receptor. Proceedings of the National Academy of Sciences of the United States of America.

[28] Sakamoto A, Kawasaki T, Kazawa T, Ohashi R, Jiang S, Maejima T, Tanaka T, Iwanari H, Hamakubo T, Sakai J, Kodama T, Naito M (2007). Expression of liver X receptor alpha in rat fetal tissues at different developmental stages. The journal of histochemistry and cytochemistry : official journal of the Histochemistry Society.

[29] Laffitte BA, Repa JJ, Joseph SB, Wilpitz DC, Kast HR, Mangelsdorf DJ, Tontonoz P (2001). LXRs control lipid-inducible expression of the apolipoprotein E gene in macrophages and adipocytes. Proceedings of the National Academy of Sciences of the United States of America.

[30] Whitney KD, Watson MA, Goodwin B, Galardi CM, Maglich JM, Wilson JG, Willson TM, Collins JL, Kliewer SA (2001). Liver X receptor (LXR) regulation of the LXRalpha gene in human macrophages. The Journal of biological chemistry.

[31] Li Y, Bolten C, Bhat BG, Woodring-Dietz J, Li S, Prayaga SK, Xia C, Lala DS (2002). Induction of human liver X receptor alpha gene expression via an autoregulatory loop mechanism. Molecular endocrinology.

[32] Rough JJ, Monroy MA, Yerrum S, Daly JM (2010). Anti-proliferative effect of LXR agonist T0901317 in ovarian carcinoma cells. Journal of ovarian research.

[33] Pommier AJ, Alves G, Viennois E, Bernard S, Communal Y, Sion B, Marceau G, Damon C, Mouzat K, Caira F, Baron S, Lobaccaro JM (2010). Liver X Receptor activation downregulates AKT survival signaling in lipid rafts and induces apoptosis of prostate cancer cells. Oncogene.

[34] Russell LE, Harrison WJ, Bahta AW, Zouboulis CC, Burrin JM, Philpott MP (2007). Characterization of liver X receptor expression and function in human skin and the pilosebaceous unit. Experimental dermatology.

[35] Fan X, Kim HJ, Bouton D, Warner M, Gustafsson JA (2008). Expression of liver X receptor beta is essential for formation of superficial cortical layers and migration of later-born neurons. Proceedings of the National Academy of Sciences of the United States of America.

[36] Modica S, Gadaleta RM, Moschetta A (2010). Deciphering the nuclear bile acid receptor FXR paradigm. Nuclear receptor signaling.

[37] Kruse MS, Rey M, Vega MC, Coirini H (2012). Alterations of LXRalpha and LXRbeta expression in the hypothalamus of glucose-intolerant rats. The Journal of endocrinology.

[38] Hong C, Tontonoz P (2014). Liver X receptors in lipid metabolism: opportunities for drug discovery. Nature reviews Drug discovery.

[39] Geyeregger R, Shehata M, Zeyda M, Kiefer FW, Stuhlmeier KM, Porpaczy E, Zlabinger GJ, Jager U, Stulnig TM (2009). Liver X receptors interfere with cytokine-induced proliferation and cell survival in normal and leukemic lymphocytes. Journal of leukocyte biology.

[40] Fukuchi J, Kokontis JM, Hiipakka RA, Chuu CP, Liao S (2004). Antiproliferative effect of liver X receptor agonists on LNCaP human prostate cancer cells. Cancer research.

[41] Vedin LL, Gustafsson JA, Steffensen KR (2013). The oxysterol receptors LXRalpha and LXRbeta suppress proliferation in the colon. Molecular carcinogenesis.

[42] Candelaria NR, Addanki S, Zheng J, Nguyen-Vu T, Karaboga H, Dey P, Gabbi C, Vedin LL, Liu K, Wu W, Jonsson PK, Lin JZ, Su F, Bollu LR, Hodges SE, McElhany AL (2014). Antiproliferative effects and mechanisms of liver X receptor ligands in pancreatic ductal adenocarcinoma cells. PloS one.

[43] Pencheva N, Buss CG, Posada J, Merghoub T, Tavazoie SF (2014). Broad-spectrum therapeutic suppression of metastatic melanoma through nuclear hormone receptor activation. Cell.

[44] Yvan-Charvet L, Pagler T, Gautier EL, Avagyan S, Siry RL, Han S, Welch CL, Wang N, Randolph GJ, Snoeck HW, Tall AR (2010). ATP-binding cassette transporters and HDL suppress hematopoietic stem cell proliferation. Science.

[45] Bensinger SJ, Bradley MN, Joseph SB, Zelcer N, Janssen EM, Hausner MA, Shih R, Parks JS, Edwards PA, Jamieson BD, Tontonoz P (2008). LXR signaling couples sterol metabolism to proliferation in the acquired immune response. Cell.

[46] Grefhorst A, Elzinga BM, Voshol PJ, Plosch T, Kok T, Bloks VW, van der Sluijs FH, Havekes LM, Romijn JA, Verkade HJ, Kuipers F (2002). Stimulation of lipogenesis by pharmacological activation of the liver X receptor leads to production of large, triglyceride-rich very low density lipoprotein particles. The Journal of biological chemistry.

[47] Joseph SB, McKilligin E, Pei L, Watson MA, Collins AR, Laffitte BA, Chen M, Noh G, Goodman J, Hagger GN, Tran J, Tippin TK, Wang X, Lusis AJ, Hsueh WA, Law RE (2002). Synthetic LXR ligand inhibits the development of atherosclerosis in mice. Proceedings of the National Academy of Sciences of the United States of America.

