# Supplementary material for: Liver X receptor reduces proliferation of human oral cancer cells by promoting cholesterol efflux via up-regulation of ABCA1 expression
Source: Oncotarget. 2015 Oct 1;6(32):33345–57. doi: 10.18632/oncotarget.5428 (PMC4741770; doi:10.18632/oncotarget.5428)
Supplement: Supplementary file 1 [file oncotarget-06-33345-s001.pdf]

SUPPLEMENTARY FIGURES

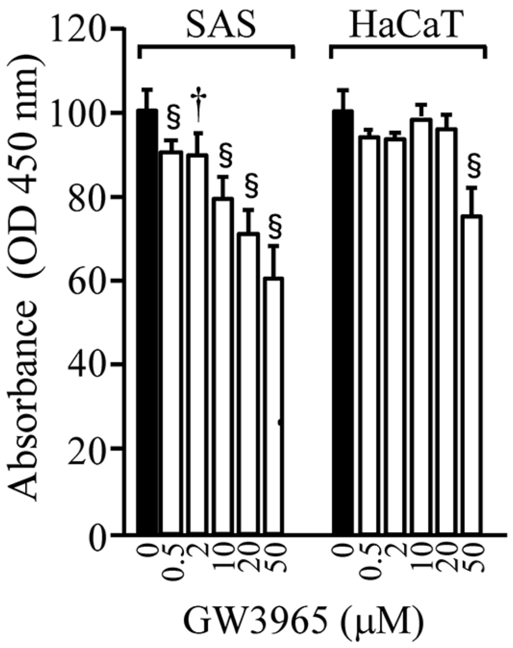

Supplementary Figure S1: The effect of GW3965 on cellular proliferation in SAS and HaCaT cells. Cells were treated for 72 h with the vehicle or the indicated concentrations of GW3965, and the viable cell numbers were determined by XTT assay. Quantification of the relative cell number is shown in the histograms, and the values represent the mean ± SD (n = 6). †$P < 0.01$, §$P < 0.001$.

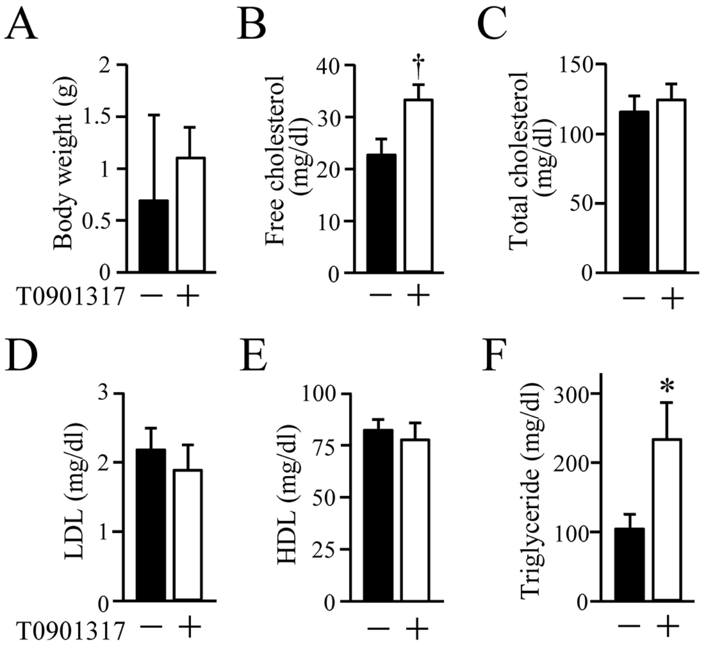

Supplementary Figure S2: The effect of T0901317 on body weight and plasma lipid content in HOSCC-transplanted mice. SCID mice were subcutaneously inoculated on their backs or chests with SAS cells, and intraperitoneally treated with vehicle (DMSO) or T0901317 (10 μg/g mice) every three days. At 15 days after the first treatment, their body weight was measured **A.** and serum obtained was subjected to further analyses **B–F.** The values represent the mean ± SD (n = 5). *$P < 0.05$, †$P < 0.01$.
